# Variation in the *MC4R* Gene Is Associated with Bone Phenotypes in Elderly Swedish Women

**DOI:** 10.1371/journal.pone.0088565

**Published:** 2014-02-06

**Authors:** Gaurav Garg, Jitender Kumar, Fiona E. McGuigan, Martin Ridderstråle, Paul Gerdhem, Holger Luthman, Kristina Åkesson

**Affiliations:** 1 Clinical and Molecular Osteoporosis Research Unit, Department of Clinical Sciences, Lund University and Department of Orthopaedics, Skåne University Hospital, Malmö, Sweden; 2 Department of Medical Sciences, Molecular Epidemiology and Science for Life Laboratory, Uppsala University, Uppsala, Sweden; 3 Clinical Obesity Research, Department of Endocrinology, Skåne University Hospital, Malmö, Sweden; 4 Department of Clinical Science, Intervention and Technology, Karolinska Institutet, Department of Orthopaedics, Karolinska University Hospital, Stockholm, Sweden; 5 Medical Genetics Unit, Department of Clinical Sciences, Lund University, Malmö, Sweden; Children’s National Medical Center, Washington, United States of America

## Abstract

Osteoporosis is characterized by reduced bone mineral density (BMD) and increased fracture risk. Fat mass is a determinant of bone strength and both phenotypes have a strong genetic component. In this study, we examined the association between obesity associated polymorphisms (SNPs) with body composition, BMD, Ultrasound (QUS), fracture and biomarkers (Homocysteine (Hcy), folate, Vitamin D and Vitamin B12) for obesity and osteoporosis. Five common variants: rs17782313 and rs1770633 (melanocortin 4 receptor *(MC4R*); rs7566605 (insulin induced gene 2 (*INSIG2*); rs9939609 and rs1121980 (fat mass and obesity associated *(FTO*) were genotyped in 2 cohorts of Swedish women: PEAK-25 (age 25, n = 1061) and OPRA (age 75, n = 1044). Body mass index (BMI), total body fat and lean mass were strongly positively correlated with QUS and BMD in both cohorts (r^2^ = 0.2–0.6). *MC4R* rs17782313 was associated with QUS in the OPRA cohort and individuals with the minor C-allele had higher values compared to T-allele homozygotes (TT vs. CT vs. CC: BUA: 100 vs. 103 vs. 103; p = 0.002); (SOS: 1521 vs. 1526 vs. 1524; p = 0.008); (Stiffness index: 69 vs. 73 vs. 74; p = 0.0006) after adjustment for confounders. They also had low folate (18 vs. 17 vs. 16; p = 0.03) and vitamin D (93 vs. 91 vs. 90; p = 0.03) and high Hcy levels (13.7 vs 14.4 vs. 14.5; p = 0.06). Fracture incidence was lower among women with the C-allele, (52% vs. 58%; p = 0.067). Variation in *MC4R* was not associated with BMD or body composition in either OPRA or PEAK-25. SNPs close to *FTO* and *INSIG2* were not associated with any bone phenotypes in either cohort and *FTO* SNPs were only associated with body composition in PEAK-25 (p≤0.001). Our results suggest that genetic variation close to *MC4R* is associated with quantitative ultrasound and risk of fracture.

## Introduction

Osteoporosis and obesity are both multifactorial disorders that in recent years have become major public health problems. At one time considered to be mutually exclusive, it is now recognized that these conditions share many genetic and environmental risk factors and are linked to each other through a number of complex regulatory pathways [1], [2]. Epidemiological studies have shown that increased body weight is positively associated with bone mass, while low body weight is a risk factor for bone loss and osteoporosis [3]. The positive effect of body weight on bone mass may be attributable to a number of factors: increased mechanical load which has an anabolic effect on bone [4]; conversion of steroid precursors to estrogen in peripheral adipose tissue [5] or through the secretion of bone active hormones from β-cells in the pancreas and adipocytes themselves [6]. Homocysteine (Hcy), vitamin B12, vitamin D and folate are biomarkers for a number of pathologies including cardiovascular disease and diabetes and a strong correlation between these biomarkers with BMI has been reported [7]–[10]. Elevated levels of Hcy and low levels of vitamin D are strong and independent risk factors for osteoporotic fracture risk [11], [12].

Studies have shown that variation in diet and life style modulate Hcy, folate and vitamin B12 [13], [14], all of which are important components in intermediary metabolism. Elevated Hcy levels have been associated with detrimental effects on bone metabolism, however, whether Hcy and other biochemical parameters play a causal role or act as markers for other mechanisms underlying these pathologies is unclear [15].

The complex relationship between fat cells and bone has been under intense scrutiny. Adipocytes and osteoblasts share a common progenitor, the pluripotent mesenchymal stem cell, and there is a degree of plasticity between the two cell types [16]–[19]. Differentiation to a particular lineage is regulated by numerous transcription factors and with increasing age there is a shift away from osteoblast towards adipocyte production [19] which in conjunction with increased osteoclast function may lead to osteoporosis [20].

At the genetic level a number of association studies have identified single nucleotide polymorphisms (SNPs) close to genes contributing to both osteoporosis and body composition [21]–[30]. To date, few bivariate genome-wide association studies (GWAS) for osteoporosis and obesity have been performed [31] although GWAS for obesity and its associated pathological outcomes have identified a number of SNPs close to genes expected to also play an important role in bone metabolism [31]. In the current study, we selected five SNPs identified through GWAS: rs17782313_*MC4R*; rs1770633_*MC4R*; rs7566605_*INSIG*2; rs9939609_*FTO* and rs1121980_*FTO*.

The *FTO* gene is highly expressed in the hypothalamus and is involved in energy homeostasis through the control of energy expenditure [32]. Located on chromosome 16, the *FTO* gene has nine exons and spans more than 400 kb. The SNPs associated with BMI in the GWAS lie in the first intron that harbors a region highly conserved across different species [33]. Individuals with the rs9939609 variant allele were shown to have a 31% increased risk per variant allele, of developing obesity [34], [35]. Variation in the *FTO* gene has been analyzed for association with BMD in a number of populations including children and adults [36] and in a mouse knockout model, BMD was lower in the *FTO* knockout compared to controls [37].

The *MC4R* gene on chromosome 18 encodes the MC4 protein, a G-protein coupled receptor that plays a major role in the central regulation of body weight through maintaining energy homeostasis and the suppression of food intake [38]. Genetic variation in the *MC4R* gene has been identified as responsible for monogenic forms of obesity [39]. In a GWAS by Loos et al., SNPs present in the intergenic region upstream of *MC4R* were associated with BMI [33]. Patients deficient in *MC4R* have been reported to have increased BMD and decreased bone resorption [40], while genetic variation has been evaluated for association with bone mass, but only in children [41].

The *INSIG2* gene has been reported to be associated with increased risk of obesity [42]. SNP rs7566605, located 10 kb upstream of the *INSIG2* gene transcription start site, was associated with fat mass. *INSIG2* is a candidate gene for increased BMI; it binds to the sterol regulatory element-binding protein complex (*SREBP)* and reduces the activity of cholesterol and fatty acid synthesis in the endoplasmic reticulum [43]. Although variation in the *INSIG2* gene has recently identified in a GWAS for BMD [44], it has not been yet fully explored.

The rationale for our study was to comprehensively evaluate the association between selected obesity associated polymorphisms and aspects of bone strength, a complex trait not captured by BMD alone. Since the skeletal fragility associated with osteoporosis reflects reduced bone quality as well as quantity, we have assessed micro-architectural properties of bone, bone geometry and long-term fracture risk in addition to BMD and body composition. Furthermore, in order to understand the mechanisms underlying these associations, we have also evaluated the association between these polymorphisms and biomarkers for obesity and bone mass. By studying two differently aged cohorts we have evaluated age-related differences in the contribution of obesity associated polymorphisms with bone phenotypes.

## Materials and Methods

### Subjects

Two population based cohorts of Swedish women were studied; the OPRA cohort consisting of 1044 elderly women all aged exactly 75 and prospectively followed for 10 years and the PEAK-25 cohort consisting of 1061 women all aged exactly 25. Details of the two cohorts have been published [45], [46]. Participants gave written informed consent and the study was approved by the Regional Ethical Review Board in Lund according to the Helsinki agreement.

### Measurement of Bone Phenotypes and Body Composition Using DXA

Bone mineral density (g/cm^2^) at the femoral neck (FN), lumbar spine (LS) and total body (TB) was measured using dual-energy x-ray absorptiometry (Lunar Prodigy: PEAK-25; Lunar DPX-L: OPRA (Lunar Corporation, Madison, WI, USA)). Fat and lean mass for total body (TB) and trunk were also measured using the same instrument. Calibrations were performed daily using a phantom supplied by the manufacturer. Precision error (coefficient of variation) for DXA scanning was 0.94%, 1.45%, 4.01% for TB, LS and FN respectively in the OPRA cohort [47] and 0.90% and 0.65% for FN and LS respectively in PEAK-25 [48]. For the OPRA cohort, all measurements at baseline were performed using the same instrument, while analyses of scans were made with software versions 1.33 and 1.35.

Hip geometry was assessed only in the OPRA cohort by employing the software provided by Lunar® (Lunar Corporation, WI, USA) for the DPX-L scanner. The following phenotypes were analyzed: Hip Axis Length (mm); femoral neck width (mm); Cross Sectional Moment of Inertia [CSMI] (Cm^4^) and Section Modulus [SM] (Cm^3^). To minimize variability, all variables were analyzed by a single operator. The coefficient of variation for these measurements was between 0.6 and 3.7% [49].

Ultrasound measurements (Speed of Sound (SOS) (m/s)), Broadband Ultrasound Attenuation ((BUA) (dB/MHz)) and Stiffness Index (SI)) were performed on the right calcaneus using the Lunar Achilles ^(R)^ system (Lunar Corporation Madison, WI, USA) to assess bone quality in both cohorts. The precision was 1.5% for derivatives of BUA and SOS [50]. Daily calibrations were made to control the long-term stability of the apparatus.

### Fracture Ascertainment

In the PEAK-25 cohort the fracture incidence is low, therefore fracture data was analyzed only in the OPRA cohort. Self-reported fractures sustained between age 20 and 75 were recorded and verified from the radiological files [51]. The majority of fractures (>99%) occurring in the elderly women were attributable to low energy trauma.

### Blood Sample Collection and Biochemical Phenotypes Measurements

Non-fasting blood samples were collected before noon for DNA isolation (PEAK-25 and OPRA) and to assay serum concentration of biochemical markers (OPRA). Samples were stored at −80°C until analysis. Assays were performed at the Department of Clinical Chemistry, Malmö, Skåne University Hospital according to accredited methods. Biochemical phenotypes, available only in the OPRA cohort, were assayed using standardized analytical protocols. Total serum Hcy (µmol/L) was measured using HPLC. Serum vitamin B12 (pmol/L) and folate (nmol/L) were measured using Elecsys assays (Roche Diagnostics, Mannheim, Germany) and serum 25-hydroxy vitamin D (25OHD) (nmol/L) was assessed by liquid chromatography mass spectrophotometry (LC-MS) [52].

### Genotyping and Statistical Analysis

Five obesity associated SNPs from three genes were genotyped in both cohorts (Table S1) [33], [42]. Sequenom’s iPlex Gold system (Sequenom, San Diego, CA) was employed to score the genotypes. A total of 993 women from the OPRA and 1001 women from the PEAK-25 cohort were genotyped successfully. Approximately 3% of the samples from each cohort were genotyped in duplicate with 100% concordance. Departures from Hardy-Weinberg equilibrium were tested using the χ^2^ test with one degree of freedom (HWE Program, Jurg Ott and Rockefeller University, New York). Linkage disequilibrium (LD) between SNPs from the same gene was tested using Haploview (http://www.broad.mit.edu/mpg/haploview/). Statistical analysis was performed using SPSS (version 20.0, SPSS Inc., Chicago, IL).

Using a co-dominant model (comparing the three genotypes, under the assumption that neither of the alleles is dominant), genotype specific differences between the phenotypes were analyzed with the Kruskal-Wallis test and to determine association adjusting for confounding factors (height, and smoking) regression analysis was performed. Gene interaction with Hcy was analyzed comparing the lowest and highest quartiles of serum Hcy levels, where quartile 1 was considered ‘Normal’ (<11.6 µmol/L) and quartile 4 ‘High’ (>17.5 µmol/L). The χ2 test was used to analyze association between genotypes and categorical variables. Multiple statistical tests were performed, however since most of the phenotypes are dependent, we report uncorrected p-values (two-tailed) and associations were considered nominally significant at the level p<0.05.

## Results

The general and clinical characteristics of the women from the two differently aged cohorts are reported in **Table 1**. Genotype and allele frequencies did not differ between cohorts (**Table 2**). All SNPs conformed to HWE (p>0.05). Both SNPs from the *FTO* gene were in strong LD (OPRA, D′ = 0.98, r^2^ = 0.84; PEAK-25, D′ = 0.99, r^2^ = 0.87) therefore only rs9939609 was used for further analysis. No LD was observed for the *MC4R* SNPs (D′ = 0.45, r^2^ = 0.13).

**Table 1:** Cohort Baseline Details.

Variable	PEAK-25	OPRA
**Age (years)**	25.5 (25.3–25.7)	75.2 (75.1–75.3)
**Weight (kg)**	63.0 (57.1–70.0)	67 (60–75)
**Height (cm)**	168 (163–172)	160 (157–164)
**BMI (kg/m^2^)**	22.4 (20.5–24.6)	26.0 (23.4–28.7)
**Smokers^#^**	440 (44%)	354 (34%)
**Adult Fracture** *	–	534 (51.1%)
**Body Composition**		
**Total Body Fat mass (kg)**	19.6 (15.2–25.1)	26.0 (20.8–31.3)
**Trunk Fat mass (kg)**	9.3 (6.9–12.2)	12.7 (9.8–15.4)
**% Fat mass- Total Body**	31.7 (26.5–36.4)	39.2 (34.1–43.1)
**% Fat mass- Trunk**	32.5 (26.2–38.9)	40.1 (35.3–44.1)
**BMD (g/cm^2^)**		
**Total Body**	1.17 (1.12–1.22)	1.00 (0.94–1.07)
**Femoral Neck**	1.04 (0.97–1.13)	0.75 (0.66–0.85)
**Lumbar Spine**	1.05 (0.99–1.13)	0.97 (0.86–1.10)
**Quantitative Ultrasound**		
**BUA (dB/MHz)**	116 (110–124)	102 (96–109)
**SOS (m/s)**	1571 (1551–1595)	1522 (1505–1540)
**Stiffness Index**	98 (88–109)	71 (62–80)
**Hip Geometry**		
**Hip Axis Length (mm)**	–	105 (102–109)
**Femoral Neck Width (mm)**	–	34 (32–36)
**Cross-Sectional Area (cm^2^)**	–	131 (114–155)
**CSMI (cm^4^)**	–	10281 (8253–13188)
**Femoral Neck Shaft** **Angle (°)**	–	129 (126–131)
**Biochemistry**		
**Homocysteine (µmol/L)**	–	14.1 (11.6–17.5)
**Vitamin B12 (pmol/L)**	–	308 (238–409)
**Folate (nmol/L)**	–	18.0 (14.0–27.0)
**Vitamin D (nmol/L)**	–	92.1 (74.3–112.2)

Median (Interquartile Range) reported for continuous variables; Number (%) for discrete variables. ^#^Current or former smokers; BUA- Broadband Ultrasound Attenuation; SOS- Speed of Sound; CSMI- Cross Sectional Moment of Inertia;

*Fracture of any type sustained after age 20 and before baseline.

**Table 2:** Genotype and Allele Frequencies.

	OPRA				PEAK-25			
SNP_Gene Symbol	Major Allele HomozygotesNo. (%)	HeterozygotesNo. (%)	Minor Allele HomozygotesNo. (%)	MAF	Major Allele HomozygotesNo. (%)	HeterozygotesNo. (%)	Minor Allele HomozygotesNo. (%)	MAF
**rs9939609_FTO**	354 (36)	499 (50)	139 (14)	0.39	319 (32)	499 (50)	182 (18)	0.43
**rs1121980_FTO**	312 (32)	500 (51)	170 (17)	0.43	282 (29)	492 (50)	211 (21)	0.47
**rs7566605_INSIG2**	448 (46)	424 (43)	111 (11)	0.33	449 (45)	421 (43)	116 (12)	0.33
**rs17782313_MC4R**	550 (57)	362 (38)	53 (5)	0.24	564 (57)	363 (37)	59 (6)	0.24
**rs17700633_MC4R**	456 (46)	439 (44)	98 (10)	0.32	482 (48)	414 (42)	98 (10)	0.31

MAF- Minor allele frequency.

The PEAK-25 participants had lower BMI and fat mass and higher lean mass compared to the elderly individuals from OPRA (**Table 1**). As previously reported, fat and lean mass were strongly positively associated with BMD [26], [53], with lean mass making a greater contribution than fat mass to BMD in young women (data not shown). For QUS phenotypes, the positive association with fat and lean mass was very similar in the elderly women, while in the young women the contribution from lean mass was stronger (**Table 3** **)**.

**Table 3:** Effect Sizes of Lean and Fat Mass on Bone Quantitative Ultrasound (QUS) Phenotypes.

		QUS Variable	
OPRA	BUA	SOS	Stiffness
**Total Body Fat mass**	0.30	0.21	0.28
**Total Body Lean** **mass**	0.32	0.22	0.28
**Trunk Fat mass**	0.32	0.23	0.31
**Trunk Lean mass**	0.31	0.21	0.30
**PEAK-25**			
**Total Body Fat mass**	0.14	−0.03^a^	0.05^a^
**Total Body Lean** **mass**	0.34	0.20	0.28
**Trunk Fat mass**	0.14	−0.02^a^	0.06^a^
**Trunk Lean mass**	0.27	0.14	0.22

Reported values are standardized β-coefficients; covariates are adjusted for height and smoking status.

All coefficients are significant at p<0.01 except for those marked ^a^ which are non-significant.

### Association between Obesity Associated Polymorphisms and Body Composition

SNP rs9939609_*FTO* was associated with a number of body composition measurements including weight, BMI, and fat mass in PEAK-25 (**Table 4**). Individuals carrying the minor allele had higher BMI, fat mass (TB and trunk) but no association was found with lean mass (**Table 4**). The association with rs9939609_*FTO* remained after adjusting for smoking status and height (p = 0.001 to p<0.0001) (**Table 4**). We observed trends for BMI, TB fat mass and percentage trunk-fat in the same direction in the OPRA cohort, but these did not reach statistical significance. After adjustment for height and smoking status an association with percentage of trunk-fat was observed with rs9939609_*FTO* (p = 0.007). No association between *MC4R* or *INSIG2* polymorphisms and body composition were observed in the OPRA and PEAK-25 cohorts (data not shown).

**Table 4:** Association of rs9939609_*FTO* with Bone and Body Composition Phenotypes in the PEAK-25 Cohort.

Phenotypes	TT	TA	AA	β-Value (Adjusted)	P-value^a^	P- value^b^
	(n = 319)	(n = 499)	(n = 182)	Co-Dominant^#^		
**Weight**	61.5 (57.0–68.7)	63.0 (57.0–69.2)	64.3 (58.0–72.3)	1.57 (0.69 to 2.44)	0.038	0.0004
**BMI**	22.1 (20.2–24.2)	22.2 (20.5–24.5)	23.0 (21.1–25.4)	0.550 (0.24 to 0.86)	0.004	0.001
**Total Body Fat mass**	19.0 (14.5–23.9)	19.4 (15.2–24.8)	21.1 (16.5–26.9)	1.36 (0.67 to 2.05)	0.004	0.0001
**Total Body Lean mass**	40.3 (37.4–43.3)	40.0 (37.1–43.1)	40.1 (37.1–43.5)	0.15 (−0.17 to 0.46)	0.63	0.36
**% Fat mass - Total Body**	30.5 (25.7–35.2)	31.7 (26.3–36.4)	32.9 (28.0–38.4)	1.17 (0.56 to 1.78)	0.002	0.0001
**Trunk Fat mass**	8.9 (6.6–11.6)	9.3 (6.9–12.1)	10.3 (7.7–13.5)	0.764 (0.39 to 1.14)	0.004	0.00007
**% Fat mass - Trunk**	31.2 (25.3–37.9)	32.5 (26.1–38.4)	34.12 (28.1–41.0)	1.36 (0.78 to 1.85)	0.002	0.0001
**Total Body BMD**	1.16 (1.12–1.22)	1.17 (1.12–1.23)	1.18 (1.13–1.22)	0.003 (−0.003 to 0.009)	0.52	0.38
**Femoral neck BMD**	1.04 (0.97–1.12)	1.04 (0.97–1.14)	1.06 (0.97–1.12)	0.003 (−0.007 to 0.014)	0.86	0.53
**Lumbar Spine BMD**	1.23 (1.15–1.31)	1.24 (1.14–1.33)	1.24 (1.14–1.33)	−0.001 (−0.012 to 0.010)	0.93	0.85
**BUA**	116 (109–124)	117 (117–123)	117 (110–124)	−0.015 (−0.977 to 0.946)	0.87	0.97
**SOS**	1569 (1552–1594)	1573 (1551–1596)	1572 (1550–1593)	−0.881 (−3.871 to 2.109)	0.60	0.56
**Stiffness Index**	96.5 (87.3–108.0)	98.0 (88.9–109.6)	99.0 (87.6–107.4)	−0.254 (−1.604 to 1.096)	0.65	0.71

Reported values are median (interquartile range); ^#^(TT vs. TA vs. AA) ^a^Kruskal-Wallis; ^b^Linear regression - after adjustment for height and smoking.

### Obesity Associated Polymorphisms and Association with BMD, QUS and Geometry

SNP rs1121980 from the *FTO* gene was excluded from analysis. The remaining four SNPs were analyzed for association with bone density, but no significant genotype related differences in BMD at any skeletal site were observed in either cohort (data not shown).

Polymorphisms were also analyzed for association with bone quantitative ultrasound in both cohorts. The rs17782313_*MC4R* showed association with BUA, SOS and SI in OPRA (p = 0.007–0.001) (**Table 5** **)** and individuals carrying the minor C-allele had higher values compared to homozygotes for the common allele. The association remained even after adjustment for height and smoking (p = 0.02–0.0004) (**Table 5**) and additional adjustment for weight (p = 0.015 to 0.007). Similar trends were observed in the PEAK-25 cohort but were not statistically significant. Polymorphisms close to *FTO* and *INSIG2* were not associated with ultrasound phenotypes in either cohort.

**Table 5:** Association of rs17782313_*MC4R* with Body Composition, Bone and Biochemistry phenotypes in the OPRA Cohort.

Phenotypes	TT	TC	CC	β-value (Adjusted)	P-value^a^	P-value^b^
	(n = 550)	(n = 362)	(n = 53)	Co-Dominant^#^		
**Weight**	66 (60–75)	68 (59–76)	67 (62–75)	0.419 (−0.703 to 1.542)	0.65	0.46
**BMI**	26.0 (23.3–28.4)	26.0 (23.5–29.1)	26.2 (23.4–27.8)	0.157 (−0.283 to 0.596)	0.58	0.49
**Total Body Fat mass**	25.8 (20.5–31.1)	26.0 (20.9–31.9)	27.1 (22.4–30.4)	0.431 (−0.419 to 1.281)	0.70	0.32
**% Fat mass Total Body**	38.5 (34.0–42.7)	39.1 (34.4–42.2)	40.0 (35.4–41.7)	0.485 (−0.276 to 1.247)	0.72	0.21
**Trunk Fat mass**	12.5 (9.8–15.3)	13.0 (9.7–15.7)	13.0 (10.4–14.8)	0.144 (−.280 to 0.568)	0.69	0.50
**% Fat mass- Trunk**	39.1 (34.2–42.7)	39.6 (34.5–43.0)	39.4 (35.8–41.9)	0.52 (−0.266 to 1.237)	0.55	0.26
**Total Body BMD**	0.997 (0.941–1.061)	1.011 (0.944–1.073)	1.003 (0.934–1.062)	0.004 (−0.006 to 0.015 )	0.52	0.44
**Femoral Neck BMD**	0.752 (0.660–0.848)	0.751 (0.680–0.846)	0.724 (0.628–0.825)	−0.005 (−0.019 to 0.009)	0.33	0.52
**Lumbar Spine BMD**	0.97 (0.86–1.10)	0.98 (0.87–1.09)	0.95 (0.84–1.12)	0.009 (−0.044 to 0.061)	0.83	0.75
**BUA**	100 (95–108)	103 (97–109)	103 (95–108)	1.72 (0.606 to 2.833)	0.007	0.002
**SOS**	1521 (1504–1539)	1526 (1509–1544)	1524 (1506–1540)	4.227 (1.119 to 7.335)	0.024	0.008
**Stiffness Index**	69.0 (61.0–79.0)	73.4 (65.0–82.5)	74.0 (61.0–81.3)	2.59 (1.11 to 4.07)	0.001	0.0006
**Homocysteine**	13.7 (11.6–16.9)	14.4 (11.6–17.7)	14.5 (11.6–18.3)	0.337 (−0.355 to 1.028)	0.06	0.34
**Folate**	18.0 (15.0–28.0)	17.0 (14.0–24.0)	16.0 (14.0–22.0)	−1.544 (−2.734 to −0.353)	0.028	0.013
**Vitamin D**	92.6 (76.7–115.5)	91.1 (72.5–109.2)	90.2 (70.3–110.5)	−3.744 (−6.914 to −0.573)	0.027	0.018

Values are median (Interquartile Range); ^#^(TT vs. TC vs. CC); ^a^Kruskal-Wallis; ^b^Linear regression after adjustment for height and smoking; Units: folate and vitamin D (nmol/L), homocysteine (µmol/L).

Femoral neck geometry is an important component of hip fracture risk and we evaluated SNP-phenotype associations in the OPRA cohort. The rs7566605_*INSIG2* showed association with FN width (p = 0.03), with individuals carrying the minor allele having a lower mean value compared to subjects homozygous for the major allele (34.1 vs. 34.5 mm), but this did not withstand adjustment for height and weight. Polymorphisms from *FTO* and *MC4R* did not show any association with bone geometry (data not shown).

### Obesity Associated Polymorphisms and Association with Biomarkers and Fracture

The association between obesity associated polymorphisms, biochemical risk factors and fracture was evaluated in the OPRA cohort. Carriers of rs17782313_*MC4R* C-allele had high Hcy (p = 0.06), low serum folate (p = 0.03) and low vitamin D (p = 0.03) (**Table 5**). After adjustment for smoking and height, the association remained for folate (p = 0.01) and vitamin D (p = 0.02).

We wanted to determine whether the association between *MC4R* SNPs and QUS was influenced in relation to normal (<11.6 µmol/L) and high (>17.5 µmol/L) levels of Hcy. Only in the high Hcy group was rs17782313_*MC4R* associated with QUS (p = 0.03 to p = 0.005) and this association remained even after adjustment for height and smoking (p = 0.007 to p = 0.001) (**Table 6**) and additionally adjusted for weight the association remained significant (p = 0.01 to 0.002). Interestingly, in the high Hcy group, vitamin D levels decreased with number of C-alleles, in direct contrast with the observation in the normal Hcy group (**Table 6**). As expected, proportionally fewer women fractured prior to baseline in the lowest (BMI<23.4; 63.9%) compared to the highest BMI quartile (BMI >28.7; 52.3%); p = 0.009).

**Table 6:** SNP *rs17782313_MC4R* Interacts with Homocysteine to Influence Bone Quality.

‘Normal’ homocysteine levels (<11.6 µmol/L) (n = 237)						
Phenotype	TT	TC	CC	β-Value (Adjusted)	p value^a^	p value^b^
	(130)	(79)	(13)	Co-Dominant#		
**Total Body Fatmass**	24.3 (18.7–29.2)	25.9 (21.1–30.4)	22.6 (19.4–28.7)	0.749 (−0.809 to 2.309)	0.35	0.35
**Folate**	32 (19–44)	29 (20–44)	32 (20–35)	−0.931 (−3.547 to 1.686)	0.76	0.48
**Vitamin D**	91.8 (77.2–115.5)	96.8 (73.7–111.0)	104 (72.3–134.0)	4.421 (−2.398 to 11.24)	0.46	0.21
**BUA**	101.4 (95.1–108.7	102 (98–109.7)	104.3 (93.0–108.7)	1.504 (−0.5546 to 3.563)	0.34	0.15
**SOS**	1522 (1505–1541)	1529 (1510–1547)	1538 (1508–1554)	3.893 (−2.348 to 10.13)	0.26	0.22
**Stiffness Index**	71 (62–80.6)	74 (65.5–83)	79 (57.6–84.7)	2.791 (−0.1968 to 5.779)	0.16	0.06
**‘High’ homocysteine levels (>17.5 µmol/L) (n = 246)**						
**Phenotype**	**TT**	**TC**	**CC**	**β-Value (Adjusted)**	**p value** a	**p value** b
	**(111)**	**(90)**	**(16)**	**Co-Dominant** #		
**Total Body Fatmass**	26.1 (19.6–31.4)	24.5 (19.2–33.2)	28.8 (24.9–30.7)	0.759 (−1.319 to 2.838)	0.49	0.48
**Folate**	14 (12–17)	14 (12–16)	14 (12–15)	−0.201 (−1.44 to 1.038)	0.87	0.75
**Vitamin D**	93.6 (75.6–116.9)	85.7 (68.9–106.7)	80.3 (64.2–104.3)	−7.394 (−13.9 to −0.8903)	0.08	0.026
**BUA**	98.7 (93.0–107.7)	103.5 (97–109)	106 (95.1–107.7)	4.356 (1.732 to 6.979)	0.006	0.001
**SOS**	1510 (1502–1535.9)	1519 (1508.2–1543)	1524 (1501–1539.9)	9.316 (2.572 to 16.06)	0.028	0.007
**Stiffness Index**	67 (59.9–77.9)	72 (64–82.5)	77 (64–76.8)	5.445 (2.188 to 8.702)	0.005	0.001

Values are median (Interquartile Range);

# (TT vs. TC vs. CC);

a Kruskal-Wallis;

b Linear regression after adjustment for height and smoking.

Variation in *FTO* and *INSIG2* did not appear to make an important contribution to fracture risk, even when smoking, TB-BMD and any one of body weight, BMI or fat mass were included in the regression model. Women carrying the *MC4R*_rs17782313 C-allele showed a non-significant trend towards fewer fractures although there was no allele dose effect (52.1% vs. 58.5%). As expected, compared to women without a baseline fracture, QUS values were lower in those with a fracture regardless of genotype (data not shown), however, within the fracture category women with the rs17782313_*MC4R* C-allele had higher QUS values ((BUA: 97 vs. 100 vs. 103; p = 0.04); (SOS: 1516 vs. 1517 vs. 1517; p = 0.18); (SI: 67 vs. 70 vs. 72; p = 0.021)) consistent with a lower fracture incidence.

## Discussion

Obesity is an established risk factor for a number of complex disorders including cardiovascular complications, diabetes mellitus and hypertension, but it has been suggested to be protective against osteoporosis [54]. A complex, differential influence from lean and fat mass on bone strength is suggested [54], [55] and the current study supports the supposition that lean mass makes a larger contribution to BMD during young age while fat mass plays a major role for BMD in later stages of life [54], [55].

*FTO* has been well described in relation to body composition and obesity phenotypes [33], [34], [36], [37]. In our study, we observed higher BMI and fat mass in relation to the rs9939609_*FTO* C-allele; however it is interesting that the association was only seen in the young women. This is consistent with suggestions that the effect size of obesity susceptibility genes varies with age [56]. Although the underlying mechanisms are unclear, data from a mouse model has shown that mRNA expression of *FTO* is regulated by nutritional intake and expression levels vary according to feeding and fasting behavior [32] and we might speculate that food intake patterns differ between young and elderly individuals. We did not find any association with BMD or other bone phenotypes in either the young or elderly cohort of women. An age-specific effect has been reported in at least one study alongside suggestions that *FTO* could be a genetic marker for peak bone mass [36] due to the potential role of *FTO* in postnatal growth [37]. Our results do not support this however since the women in the PEAK-25 cohort are at an age where peak bone mass is assumed to have been reached. Nonetheless this does not rule out the possibility that *FTO* variants could be associated with skeletal growth trajectory in childhood and adolescence. Although we found no direct association between variations in the vicinity of *FTO* and BMD or QUS parameters, it is likely that any effect of the gene on bone is indirect, through BMI and fat mass.

*MC4R* is crucial in the regulation of body weight and monogenic forms of obesity commonly result from mutations in its gene. Although we observed a trend for higher BMI and fat mass in both cohorts with the C-allele, the association with *MC4R* did not reach significance, which contrasts with the findings reported in GWAS [33] and other association studies [57]. In the current study, we have shown for the first time that variation in the *MC4R* gene is associated with QUS phenotypes. A trend towards better bone quality with carriage of the variant *MC4R* rs17782313 C-allele was observed in the young women, but was more pronounced in the elderly women. Furthermore this association appeared to be mediated by both direct and indirect mechanisms which may explain in part the age specific effect observed, since a higher BMI, as displayed by the older women, is positively associated with bone strength. Although a genetic association between *MC4R* gene polymorphism and bone mass has been reported, albeit in children [41], we found no association with BMD or bone structural traits (femoral neck geometry) in our study.

One of the novel findings of our study is that *MC4R* is associated with altered vitamin D, folate and Hcy levels, which are associated with obesity. Vitamin D deficiency associated with obesity has been shown at all ages and independent of sex in a recent meta-analysis [58]. The results from our study indicates that a gene environment interaction has the potential to improve bone quality through increased fat mass in elderly women, demonstrated by the fact that the strongest association between *MC4R* and QUS was in the elevated Hcy group. This finding is in keeping with what is known about *MC4R* expression, i.e. that it is altered in response to environmental stimuli through hypothalamic neuronal networks, and recent studies suggest this has an important role in bone homeostasis [59].

Although in a meta-analysis of GWAS [39] variation in *INSIG2* was associated with femoral neck BMD, in our cohorts *INSIG2* was not associated with BMD, body composition or bone quality, although this is unsurprising since we have analyzed a BMI associated SNP which is not in LD (r^2^ = 0.01; D′ = 0.39) with the SNP identified in the BMD GWAS. In our study, although width at the femoral neck was narrower in elderly women with the variant allele, the association was attenuated after adjustment for height and weight and furthermore indices of bone strength and hip fracture rates were not different. To date, none of the GWAS for bone geometry have shown evidence of association within or near the *INSIG2* gene [60]–[63].

The strengths of this study include the extensive data collected on body composition, bone related phenotypes and biochemical risk factors for obesity and osteoporosis. By including two differently aged cohorts we have the possibility to distinguish age related effects of genetic variation on these phenotypes. The cohorts studied are well-characterized, large, of identical age within each cohort and the majority of women were of Swedish origin. Whether the findings are applicable to other ethnic groups requires replication in other populations. A limitation of the study is that biomarker data was not available in the PEAK-25 cohort, which would have enabled us to identify if there are age related effects associated with the homocysteine-*MC4R*-obesity relationship.

In summary, our data provides novel evidence that variation in the obesity associated gene *MC4R* is associated with improved quantitative ultrasound phenotypes, an important component of bone strength.

## Supporting Information

Table S1 **Obesity Related Gene Polymorphisms Studied in the PEAK-25 and OPRA Cohorts.** FTO-Fat mass and Obesity- associated protein; INSIG2- Insulin induced gene 2; MC4R- Melanocortin receptor 4.
(DOCX)

## References

[1] Deng FY, Lei SF, Li MX, Jiang C, Dvornyk V, et al (2006) Genetic determination and correlation of body mass index and bone mineral density at the spine and hip in Chinese Han ethnicity. Osteoporos Int 17: 119–124. 16025191 10.1007/s00198-005-1930-4

[2] Sun X, Lei SF, Deng FY, Wu S, Papacian C, et al (2006) Genetic and environmental correlations between bone geometric parameters and body compositions. Calcif Tissue Int 79: 43–49. 16868663 10.1007/s00223-006-0041-3

[3] Wardlaw GM (1996) Putting body weight and osteoporosis into perspective. Am J Clin Nutr 63: 433S–436S. 8615336 10.1093/ajcn/63.3.433

[4] Skerry TM, Suva LJ (2003) Investigation of the regulation of bone mass by mechanical loading: from quantitative cytochemistry to gene array. Cell Biochem Funct 21: 223–229. 12910474 10.1002/cbf.1077

[5] Horowitz MC (1993) Cytokines and estrogen in bone: anti-osteoporotic effects. Science 260: 626–627. 8480174 10.1126/science.8480174

[6] Reid IR (2002) Relationships among body mass, its components, and bone. Bone 31: 547–555. 12477567 10.1016/s8756-3282(02)00864-5

[7] Nakazato M, Maeda T, Takamura N, Wada M, Yamasaki H, et al (2011) Relation of body mass index to blood folate and total homocysteine concentrations in Japanese adults. Eur J Nutr 50: 581–585. 21221977 10.1007/s00394-010-0165-0

[8] Vaya A, Ejarque I, Tembl J, Corella D, Laiz B (2011) Hyperhomocysteinemia, obesity and cryptogenic stroke. Clin Hemorheol Microcirc 47: 53–58. 21321408 10.3233/CH-2010-1365

[9] Vaya A, Rivera L, Hernandez-Mijares A, de la Fuente M, Sola E, et al (2012) Homocysteine levels in morbidly obese patients: its association with waist circumference and insulin resistance. Clin Hemorheol Microcirc 52: 49–56. 22460264 10.3233/CH-2012-1544

[10] Jungert A, Roth HJ, Neuhauser-Berthold M (2012) Serum 25-hydroxyvitamin D3 and body composition in an elderly cohort from Germany: a cross-sectional study. Nutr Metab (Lond) 9: 42. 22607088 10.1186/1743-7075-9-42 PMC3484027

[11] Ensrud KE, Ewing SK, Fredman L, Hochberg MC, Cauley JA, et al (2010) Circulating 25-hydroxyvitamin D levels and frailty status in older women. J Clin Endocrinol Metab 95: 5266–5273. 21131545 10.1210/jc.2010-2317 PMC2999979

[12] van Meurs JB, Dhonukshe-Rutten RA, Pluijm SM, van der Klift M, de Jonge R, et al (2004) Homocysteine levels and the risk of osteoporotic fracture. N Engl J Med 350: 2033–2041. 15141041 10.1056/NEJMoa032546

[13] McLean RR, Jacques PF, Selhub J, Tucker KL, Samelson EJ, et al (2004) Homocysteine as a predictive factor for hip fracture in older persons. N Engl J Med 350: 2042–2049. 15141042 10.1056/NEJMoa032739

[14] Morris MS, Jacques PF, Selhub J (2005) Relation between homocysteine and B-vitamin status indicators and bone mineral density in older Americans. Bone 37: 234–242. 15950558 10.1016/j.bone.2005.04.017

[15] Vacek TP, Kalani A, Voor MJ, Tyagi SC, Tyagi N (2013) The role of homocysteine in bone remodeling. Clin Chem Lab Med 51: 579–590. 23449525 10.1515/cclm-2012-0605 PMC3951268

[16] Bellows CG, Heersche JN (2001) The frequency of common progenitors for adipocytes and osteoblasts and of committed and restricted adipocyte and osteoblast progenitors in fetal rat calvaria cell populations. J Bone Miner Res 16: 1983–1993. 11697794 10.1359/jbmr.2001.16.11.1983

[17] Beresford JN, Bennett JH, Devlin C, Leboy PS, Owen ME (1992) Evidence for an inverse relationship between the differentiation of adipocytic and osteogenic cells in rat marrow stromal cell cultures. J Cell Sci 102 (Pt 2): 341–351. 10.1242/jcs.102.2.341 1400636

[18] Carnevale V, Romagnoli E, Del Fiacco R, Pepe J, Cipriani C, et al (2010) Relationship between bone metabolism and adipogenesis. J Endocrinol Invest 33: 4–8. 20938218

[19] Gimble JM, Robinson CE, Wu X, Kelly KA (1996) The function of adipocytes in the bone marrow stroma: an update. Bone 19: 421–428. 8922639 10.1016/s8756-3282(96)00258-x

[20] Rosen CJ, Bouxsein ML (2006) Mechanisms of disease: is osteoporosis the obesity of bone? Nat Clin Pract Rheumatol 2: 35–43. 16932650 10.1038/ncprheum0070

[21] Ackert-Bicknell CL, Demissie S, Marin de Evsikova C, Hsu YH, DeMambro VE, et al (2008) PPARG by dietary fat interaction influences bone mass in mice and humans. J Bone Miner Res 23: 1398–1408. 18707223 10.1359/JBMR.080419 PMC2683155

[22] Bustamante M, Nogues X, Mellibovsky L, Agueda L, Jurado S, et al (2007) Polymorphisms in the interleukin-6 receptor gene are associated with bone mineral density and body mass index in Spanish postmenopausal women. Eur J Endocrinol 157: 677–684. 17984249 10.1530/EJE-07-0389

[23] Cha S, Yu H, Kim JY (2012) Bone mineral density-associated polymorphisms are associated with obesity-related traits in Korean adults in a sex-dependent manner. PLoS One 7: e53013. 23300848 10.1371/journal.pone.0053013 PMC3531417

[24] Fairbrother UL, Tanko LB, Walley AJ, Christiansen C, Froguel P, et al (2007) Leptin receptor genotype at Gln223Arg is associated with body composition, BMD, and vertebral fracture in postmenopausal Danish women. J Bone Miner Res 22: 544–550. 17243864 10.1359/jbmr.070114

[25] McGuigan F, Larzenius E, Callreus M, Gerdhem P, Luthman H, et al (2008) Variation in the bone morphogenetic protein-2 gene: effects on fat and lean body mass in young and elderly women. Eur J Endocrinol 158: 661–668. 18426824 10.1530/EJE-07-0757

[26] McGuigan FE, Larzenius E, Callreus M, Gerdhem P, Luthman H, et al (2007) Variation in the BMP2 gene: bone mineral density and ultrasound in young adult and elderly women. Calcif Tissue Int 81: 254–262. 17726567 10.1007/s00223-007-9054-9

[27] Piters E, de Freitas F, Nielsen TL, Andersen M, Brixen K, et al (2012) Association study of polymorphisms in the SOST gene region and parameters of bone strength and body composition in both young and elderly men: data from the Odense Androgen Study. Calcif Tissue Int 90: 30–39. 22076526 10.1007/s00223-011-9546-5

[28] Xiao WJ, He JW, Zhang H, Hu WW, Gu JM, et al (2011) ALOX12 polymorphisms are associated with fat mass but not peak bone mineral density in Chinese nuclear families. Int J Obes (Lond) 35: 378–386. 20697415 10.1038/ijo.2010.157 PMC3061002

[29] Zhao J, Bradfield JP, Li M, Zhang H, Mentch FD, et al (2011) BMD-associated variation at the Osterix locus is correlated with childhood obesity in females. Obesity (Silver Spring) 19: 1311–1314. 21212767 10.1038/oby.2010.324

[30] Zhao LJ, Guo YF, Xiong DH, Xiao P, Recker RR, et al (2006) Is a gene important for bone resorption a candidate for obesity? An association and linkage study on the RANK (receptor activator of nuclear factor-kappaB) gene in a large Caucasian sample. Hum Genet 120: 561–570. 16960694 10.1007/s00439-006-0243-9 PMC1829481

[31] Liu YZ, Pei YF, Liu JF, Yang F, Guo Y, et al (2009) Powerful bivariate genome-wide association analyses suggest the SOX6 gene influencing both obesity and osteoporosis phenotypes in males. PLoS One 4: e6827. 19714249 10.1371/journal.pone.0006827 PMC2730014

[32] Gerken T, Girard CA, Tung YC, Webby CJ, Saudek V, et al (2007) The obesity-associated FTO gene encodes a 2-oxoglutarate-dependent nucleic acid demethylase. Science 318: 1469–1472. 17991826 10.1126/science.1151710 PMC2668859

[33] Loos RJ, Lindgren CM, Li S, Wheeler E, Zhao JH, et al (2008) Common variants near MC4R are associated with fat mass, weight and risk of obesity. Nat Genet 40: 768–775. 18454148 10.1038/ng.140 PMC2669167

[34] Frayling TM, Timpson NJ, Weedon MN, Zeggini E, Freathy RM, et al (2007) A common variant in the FTO gene is associated with body mass index and predisposes to childhood and adult obesity. Science 316: 889–894. 17434869 10.1126/science.1141634 PMC2646098

[35] Rouskas K, Kouvatsi A, Paletas K, Papazoglou D, Tsapas A, et al (2012) Common variants in FTO, MC4R, TMEM18, PRL, AIF1, and PCSK1 show evidence of association with adult obesity in the Greek population. Obesity (Silver Spring) 20: 389–395. 21720444 10.1038/oby.2011.177

[36] Guo Y, Liu H, Yang TL, Li SM, Li SK, et al (2011) The fat mass and obesity associated gene, FTO, is also associated with osteoporosis phenotypes. PLoS One 6: e27312. 22125610 10.1371/journal.pone.0027312 PMC3220685

[37] Gao X, Shin YH, Li M, Wang F, Tong Q, et al (2010) The fat mass and obesity associated gene FTO functions in the brain to regulate postnatal growth in mice. PLoS One 5: e14005. 21103374 10.1371/journal.pone.0014005 PMC2982835

[38] Tao YX (2010) The melanocortin-4 receptor: physiology, pharmacology, and pathophysiology. Endocr Rev 31: 506–543. 20190196 10.1210/er.2009-0037 PMC3365848

[39] Willer CJ, Speliotes EK, Loos RJ, Li S, Lindgren CM, et al (2009) Six new loci associated with body mass index highlight a neuronal influence on body weight regulation. Nat Genet 41: 25–34. 19079261 10.1038/ng.287 PMC2695662

[40] Ahn JD, Dubern B, Lubrano-Berthelier C, Clement K, Karsenty G (2006) Cart overexpression is the only identifiable cause of high bone mass in melanocortin 4 receptor deficiency. Endocrinology 147: 3196–3202. 16614075 10.1210/en.2006-0281

[41] Timpson NJ, Sayers A, Davey-Smith G, Tobias JH (2009) How does body fat influence bone mass in childhood? A Mendelian randomization approach. J Bone Miner Res 24: 522–533. 19016587 10.1359/jbmr.081109 PMC2875165

[42] Herbert A, Gerry NP, McQueen MB, Heid IM, Pfeufer A, et al (2006) A common genetic variant is associated with adult and childhood obesity. Science 312: 279–283. 16614226 10.1126/science.1124779

[43] Gong Y, Lee JN, Brown MS, Goldstein JL, Ye J (2006) Juxtamembranous aspartic acid in Insig-1 and Insig-2 is required for cholesterol homeostasis. Proc Natl Acad Sci U S A 103: 6154–6159. 16606821 10.1073/pnas.0601923103 PMC1435366

[44] Estrada K, Styrkarsdottir U, Evangelou E, Hsu YH, Duncan EL, et al (2012) Genome-wide meta-analysis identifies 56 bone mineral density loci and reveals 14 loci associated with risk of fracture. Nat Genet 44: 491–501. 22504420 10.1038/ng.2249 PMC3338864

[45] Gerdhem P, Brandstrom H, Stiger F, Obrant K, Melhus H, et al (2004) Association of the collagen type 1 (COL1A 1) Sp1 binding site polymorphism to femoral neck bone mineral density and wrist fracture in 1044 elderly Swedish women. Calcif Tissue Int 74: 264–269. 14595528 10.1007/s00223-002-2159-2

[46] Kumar J, Swanberg M, McGuigan F, Callreus M, Gerdhem P, et al (2011) LRP4 association to bone properties and fracture and interaction with genes in the Wnt- and BMP signaling pathways. Bone 49: 343–348. 21645651 10.1016/j.bone.2011.05.018

[47] Lenora J, Akesson K, Gerdhem P (2010) Effect of precision on longitudinal follow-up of bone mineral density measurements in elderly women and men. J Clin Densitom 13: 407–412. 20605500 10.1016/j.jocd.2010.04.004

[48] Callreus M, McGuigan F, Ringsberg K, Akesson K (2012) Self-reported recreational exercise combining regularity and impact is necessary to maximize bone mineral density in young adult women: a population-based study of 1,061 women 25 years of age. Osteoporos Int 23: 2517–2526. 22246601 10.1007/s00198-011-1886-5

[49] Tenne M, McGuigan FE, Ahlborg H, Gerdhem P, Akesson K (2010) Variation in the PTH gene, hip fracture, and femoral neck geometry in elderly women. Calcif Tissue Int 86: 359–366. 20349051 10.1007/s00223-010-9351-6

[50] Karlsson MK, Obrant KJ, Nilsson BE, Johnell O (1998) Bone mineral density assessed by quantitative ultrasound and dual energy X-ray absorptiometry. Normative data in Malmo, Sweden. Acta Orthop Scand 69: 189–193. 9602782 10.3109/17453679809117626

[51] Gerdhem P, Akesson K (2007) Rates of fracture in participants and non-participants in the Osteoporosis Prospective Risk Assessment study. J Bone Joint Surg Br 89: 1627–1631. 18057364 10.1302/0301-620X.89B12.18946

[52] Gerdhem P, Ivaska KK, Isaksson A, Pettersson K, Vaananen HK, et al (2007) Associations between homocysteine, bone turnover, BMD, mortality, and fracture risk in elderly women. J Bone Miner Res 22: 127–134. 17032146 10.1359/jbmr.061003

[53] Gerdhem P, Ringsberg KA, Akesson K, Obrant KJ (2003) Influence of muscle strength, physical activity and weight on bone mass in a population-based sample of 1004 elderly women. Osteoporos Int 14: 768–772. 12904841 10.1007/s00198-003-1444-x

[54] Namwongprom S, Rojanasthien S, Mangklabruks A, Soontrapa S, Wongboontan C, et al (2013) Effect of fat mass and lean mass on bone mineral density in postmenopausal and perimenopausal Thai women. Int J Womens Health 5: 87–92. 23467695 10.2147/IJWH.S41884 PMC3589079

[55] Hawamdeh ZM, Sheikh-Ali RF, Alsharif A, Otom AH, Ibrahim AI, et al.. (2013) The Influence of Aging on the Association Between Adiposity and Bone Mineral Density in Jordanian Postmenopausal Women. J Clin Densitom. [Epub ahead of print]. 10.1016/j.jocd.2013.02.007 23499561

[56] Kvaloy K, Kulle B, Romundstad P, Holmen TL (2013) Sex-specific effects of weight-affecting gene variants in a life course perspective-The HUNT Study, Norway. Int J Obes (Lond) 9: 1221–9. 10.1038/ijo.2012.220 23318717

[57] Liem ET, Vonk JM, Sauer PJ, van der Steege G, Oosterom E, et al (2010) Influence of common variants near INSIG2, in FTO, and near MC4R genes on overweight and the metabolic profile in adolescence: the TRAILS (TRacking Adolescents’ Individual Lives Survey) Study. Am J Clin Nutr 91: 321–328. 20007308 10.3945/ajcn.2009.28186

[58] Vimaleswaran KS, Berry DJ, Lu C, Tikkanen E, Pilz S, et al (2013) Causal relationship between obesity and vitamin D status: bi-directional Mendelian randomization analysis of multiple cohorts. PLoS Med 10: e1001383. 23393431 10.1371/journal.pmed.1001383 PMC3564800

[59] Patel MS, Elefteriou F (2007) The new field of neuroskeletal biology. Calcif Tissue Int 80: 337–347. 17440766 10.1007/s00223-007-9015-3

[60] Chen Y, Xiong DH, Guo YF, Pan F, Zhou Q, et al (2010) Pathway-based genome-wide association analysis identified the importance of EphrinA-EphR pathway for femoral neck bone geometry. Bone 46: 129–136. 19786129 10.1016/j.bone.2009.09.025 PMC2818219

[61] Hsu YH, Zillikens MC, Wilson SG, Farber CR, Demissie S, et al (2010) An integration of genome-wide association study and gene expression profiling to prioritize the discovery of novel susceptibility Loci for osteoporosis-related traits. PLoS Genet 6: e1000977. 20548944 10.1371/journal.pgen.1000977 PMC2883588

[62] Liu YZ, Wilson SG, Wang L, Liu XG, Guo YF, et al (2008) Identification of PLCL1 gene for hip bone size variation in females in a genome-wide association study. PLoS One 3: e3160. 18776929 10.1371/journal.pone.0003160 PMC2522269

[63] Zhao LJ, Liu XG, Liu YZ, Liu YJ, Papasian CJ, et al (2010) Genome-wide association study for femoral neck bone geometry. J Bone Miner Res 25: 320–329. 20175129 10.1359/jbmr.090726 PMC3153387

