# Supplementary material for: Variation in the MC4R Gene Is Associated with Bone Phenotypes in Elderly Swedish Women
Source: PLoS One. 2014 Feb 6;9(2):e88565. doi: 10.1371/journal.pone.0088565 (PMC3916440; doi:10.1371/journal.pone.0088565)
Supplement: Table S1 — Obesity Related Gene Polymorphisms Studied in the PEAK-25 and OPRA Cohorts. FTO-Fat mass and Obesity- associated protein; INSIG2- Insulin induced gene 2; MC4R- Melanocortin receptor 4. (DOCX) [file pone.0088565.s001.docx]

**Table S1:** Obesity Related Gene Polymorphisms Studied In the PEAK-25 and OPRA Cohorts

| **rs ID** | **Closest Gene** | **Chr No (Position)** | **Base Change** | **Location** |
| --- | --- | --- | --- | --- |
| rs9939609 | *FTO* | 16 (53820527) | T/A | Intron 1 |
| rs1121980 | *FTO* | 16 (53809247) | G/A | Intron 1 |
| rs7566605 | *INSIG2* | 2 (118836025) | G/C | Intergenic |
| rs17782313 | *MC4R* | 18 (57851097) | T/C | Intergenic |
| rs17700633 | *MC4R* | 18 (57929432) | G/A | Intergenic |
